# Integrating PBPK Simulation and In Vitro Pharmacodynamics to Model Efavirenz-Associated Hepatic Oxidative Stress in Pregnancy

**DOI:** 10.3390/pharmaceutics18101253

**Published:** 2026-10-02

**Authors:** Bárbara Costa, Maria João Gouveia, Nuno Vale

**Affiliations:** 1 PerMed Research Group, RISE-Health, Faculty of Medicine, University of Porto, Alameda Professor Hernâni Monteiro, 4200-319 Porto, Portugal; bcosta@med.up.pt; 2 RISE-Health, Department of Community Medicine, Health Information and Decision (MEDCIDS), Faculty of Medicine, University of Porto, Rua Doutor Plácido da Costa, 4200-450 Porto, Portugal; 3 Centre for the Study in Animal Science, Medical and Biomedical Sciences School, Fernando Pessoa University, 4420-096 Gondomar, Portugal; 4 Centre for Parasite Biology and Immunology, Department of Infectious Diseases, National Health Institute Dr. Ricardo Jorge, 4000-055 Porto, Portugal; 5 Laboratory of Personalized Medicine, Department of Community Medicine, Health Information and Decision (MEDCIDS), Faculty of Medicine, University of Porto, Rua Doutor Plácido da Costa, 4200-450 Porto, Portugal

**Keywords:** efavirenz, PBPK-PD modelling, oxidative stress, pregnancy pharmacokinetics, CYP2B6 polymorphism, QIVIVE, translational pharmacology, antiretroviral therapy

## Abstract

**Background**: Certain antiretroviral drugs, including efavirenz, have been associated with mitochondrial and oxidative effects in hepatic cells, but quantitative translation from nominal in vitro concentrations to an in vivo effect-site concentration remains uncertain. **Methods**: A hypothesis-generating physiologically based pharmacokinetic-pharmacodynamic (PBPK-PD) framework integrated simulated EFV concentration-time profiles for six pregnancy/CYP2B6 scenarios with published Hep3B MitoSOX concentration-response data fitted with the Hill–Emax model. Translational exposure was evaluated as total hepatic EFV concentration relative to the nominal in vitro concentration range and as unbound hepatic concentration approximated from total plasma concentration and fraction unbound. **Results**: Simulations predicted lower total plasma exposure during pregnancy in the reference normal-metaboliser scenario (C_p,total_ Cmax: decreasing from 9.94 to 7.15 µM). Under the free-drug scenario (f_u,p_ = 0.22%), the estimated Cmax,u decreased from 0.0219 to 0.0157 µM, remaining far below the lowest non-zero nominal in vitro concentration (10 µM). Benchmarked against the fu,vitro-corrected in vitro EC_50_ (0.324 μM via Kalvass–Maurer protein-binding correction), this margin narrowed to approximately 15- to 21-fold at the reference f_u,p_ and to approximately 2-fold at the highest literature-reported value of f_u,p_ (1.5%). **Conclusions**: Pregnancy and CYP2B6 phenotype altered simulated EFV exposure, whereas quantitative translation to the Hep3B oxidative stress response depended strongly on the concentration metric used. Because unbound EFV was not reported in the in vitro system and intracellular hepatic EFV was unavailable, neither comparison establishes a definitive effect-site match. This framework supports an exploratory QIVIVE interpretation and identifies the exposure measurements required for quantitative validation.

## 1. Introduction

Antiretroviral therapy (ART) during pregnancy is essential to preserve maternal health and reduce the risk of vertical HIV transmission. Because this treatment is taken over a prolonged period that overlaps active foetal development, any drug-related risk to the foetus must be weighed against its clear maternal benefit [1]. In this context, certain regimens have been associated with higher rates of preterm birth, low birth weight, and small-for-gestational-age infants, though these associations must be interpreted in the context of HIV disease severity, the timing of treatment, and regimen type, all of which strongly influence perinatal risk [2]. The biological mechanisms underlying this regimen-specific risk are not fully established, but mitochondrial dysfunction and oxidative stress recur as concerns in the ART literature, particularly for nucleoside reverse transcriptase inhibitor (NRTI)-containing regimens, though not exclusively [3,4]. Clinical studies have reported markers consistent with increased oxidative stress after ART initiation, including higher lipid peroxidation, reduced antioxidant defences, and greater DNA oxidative damage, and studies in HIV/ART-exposed pregnancies and infants have described altered mitochondrial DNA content, changes in enzyme expression, and placental oxidative stress markers [5,6,7,8,9]. Whether oxidative stress causally contributes to the adverse perinatal outcomes seen in ART-exposed pregnancies is still unknown, but it remains a plausible pathway given its established role in placental dysfunction, foetal growth restriction, and preterm birth [10,11,12].

Translating this plausibility into drug-specific inference is constrained by the available methods. Clinical biomarkers such as F2-isoprostanes and 8-hydroxydeoxyguanosine give indirect systemic measures but do not capture tissue-specific or intracellular events [13], whereas in vitro models can directly assess mitochondrial ROS production, membrane potential, and bioenergetic disruption, but they are typically run at fixed concentrations and therefore do not reproduce the concentration-time profiles of clinical dosing [14,15]. Neither approach alone can determine whether an in vitro concentration-dependent oxidative stress signal is pharmacodynamically relevant at the exposures patients actually experience; that requires linking biomarker data, in vitro potency, and clinical pharmacokinetics. Pregnancy compounds this, since changes in hepatic blood flow, enzyme activity, and plasma volume alter drug disposition in ways that vary by drug and genotype, so maternal exposure can differ substantially from that of non-pregnant and pregnant adults [16]. Closing this gap requires a framework that links pregnancy-specific pharmacokinetic predictions to a quantitative in vitro pharmacodynamic surface.

Efavirenz is a useful case for this approach because it remains in clinical use in high-burden settings, and its exposure is strongly genotype-dependent due to CYP2B6-mediated hepatic clearance; pregnancy-related physiological changes may further modify its pharmacokinetics [17]. Pharmacokinetic and modelling studies show that pregnancy generally reduces EFV exposure through increased clearance, but genotype remains the dominant source of between-person variability and can produce exposure differences larger than the average pregnancy effect [18,19]. Physiologically based pharmacokinetic (PBPK) models have been used to quantify these shifts in maternal compartments and to support dose evaluation for antiretroviral drugs in pregnancy, providing a validated way to generate tissue-level concentration-time predictions when clinical data are sparse [20,21]. Coupling such predictions to an in vitro pharmacodynamic model fitted to hepatic oxidative stress data allows a formal test of whether pregnancy- and genotype-driven exposure changes are large enough to shift the modelled pharmacodynamic burden under a fixed concentration-response surface [22,23,24]. This cannot replace a clinical result, but it can show whether the pharmacokinetic differences are pharmacodynamically consequential within the model’s assumptions and identify the conditions under which they are.

**Figure 1:**
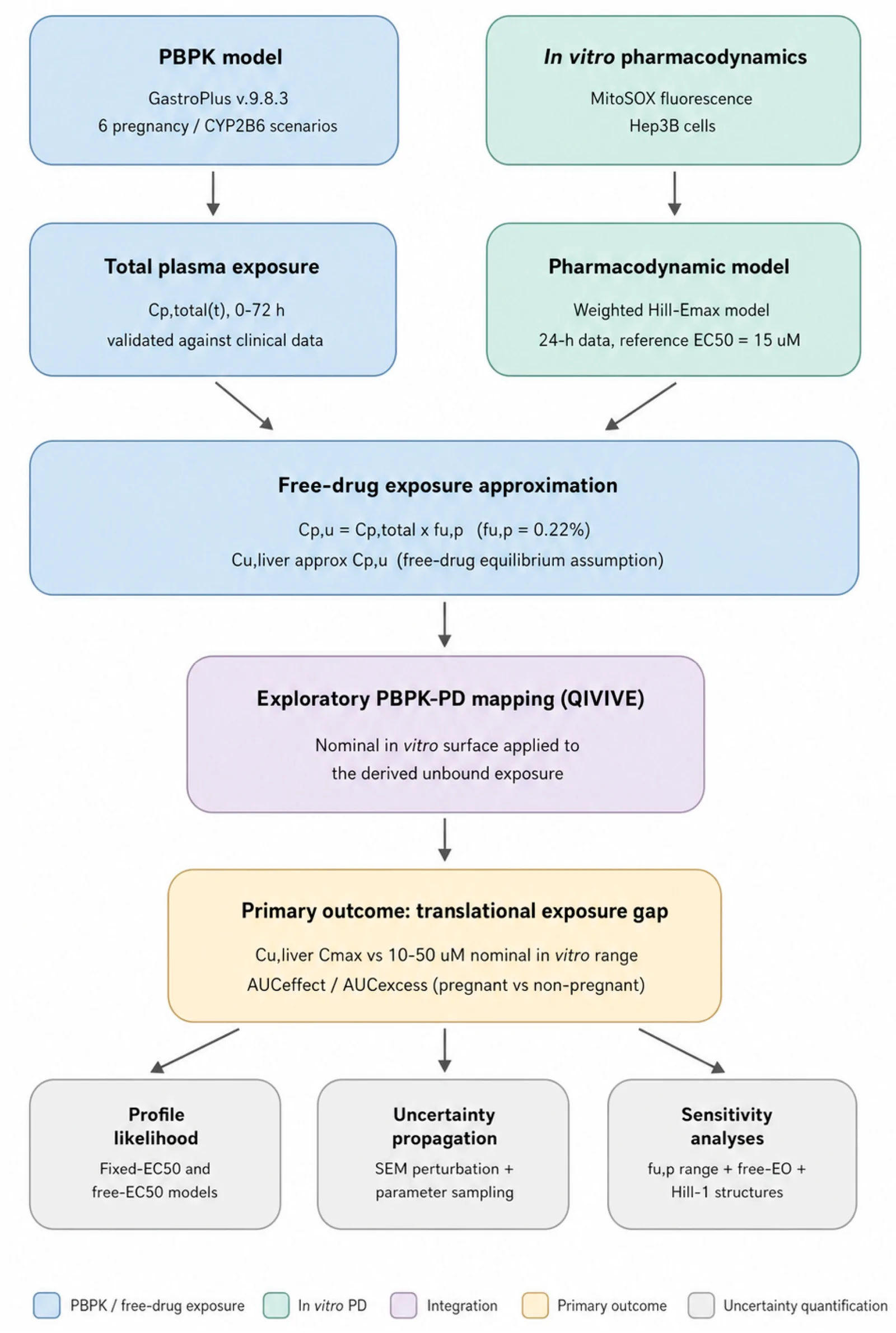
Analytical workflow linking the published Hep3B MitoSOX concentration-response data to PBPK-predicted EFV exposure. The 24 h in vitro concentration-response relationship was used as the primary pharmacodynamic anchor. Total venous plasma profiles were simulated for six pregnancy/CYP2B6 scenarios over 72 h; unbound plasma exposure was derived using f_u,p_ as an explicit free-drug approximation of unbound hepatic exposure. This exposure was mapped onto the fitted in vitro concentration–response relationshipto define the primary outcome, the translational exposure gap and AUCeffect/AUCexcess ratios between pregnant and non-pregnant scenarios, whose robustness was assessed through profile-likelihood, uncertainty propagation, and sensitivity analyses.

The objective of this study was to develop a hypothesis-generating translational PBPK-PD framework integrating simulated EFV exposure across pregnancy status and CYP2B6 phenotype with published Hep3B MitoSOX concentration-response data. In Figure 1, we demonstrate the analytical workflow used to investigate whether pregnancy- and genotype-dependent differences in EFV exposure could be related to the in vitro oxidative stress response while accounting for uncertainty in the concentration available at the effect site.

## 2. Methods

### 2.1. Study Design and Analytical Framework

This study used a quantitative, model-based translational framework. No human participants or animals were involved. The analysis combined two pre-existing data sources, GastroPlus-derived pharmacokinetic simulations and published in vitro concentration-response data and integrated them computationally to generate predicted pharmacodynamic effect-time profiles (GastroPlus v9.9, Simulation Plus Inc., Lancaster, CA, USA). The study does not involve primary data collection from human subjects and did not require ethical approval. All analytical procedures were implemented in R (version 4.5.3) using the packages ggplot2, DescTools, patchwork, dplyr, tidyr, readxl, and MASS (accessed via explicit namespace qualification (MASS::mvrnorm) rather than attached directly, to avoid a documented conflict with dplyr::select()). A random seed was set at the start of the analysis to ensure reproducibility of all stochastic procedures. Code and intermediate output files are provided as Supplementary Material. During manuscript preparation, generative AI was used in a supporting role to cross-check statistical results with the code, confirm important references, and help draft and refine some sections of the text.

### 2.2. Physiologically Based Pharmacokinetic Model and Simulated Hepatic Profiles

Plasma and hepatic efavirenz (EFV) concentration-time profiles were generated using GastroPlus v.9.8.3 (Simulation Plus Inc., Lancaster, CA, USA) to simulate six physiological scenarios, representing all combinations of pregnancy status and CYP2B6 metaboliser phenotype. The plasma profiles were used to validate the model against values reported in the literature, whereas the hepatic profiles provided the tissue-level exposures used to drive the pharmacodynamic model. The PBPK model was based on a previously developed and partially validated EFV model derived from Simulation Plus compound library parameters [25], which was also used in a prior pharmacokinetic modelling study by this group [26]. Model construction and validation against the existing literature are described in those references and are not repeated here; the same structural model was used without re-estimation of compound-specific parameters. Key physicochemical and biopharmaceutical parameters are provided in Supplementary Table S1. Tissue-to-plasma partition coefficients were predicted using the Lukacova method under a perfusion-limited distribution assumption for all tissues [21].

It should be noted that predictive performance for the pregnant EFV scenarios in the prior validation work was below the prespecified 25% prediction error threshold for AUC, which was attributed to the absence of genotype stratification in the available clinical data [26]. The simulated hepatic profiles for the pregnant scenarios therefore carry greater pharmacokinetic uncertainty than those for the non-pregnant adult, and this is acknowledged as a limitation of the current analysis.

Six physiological scenarios were simulated, representing all combinations of pregnancy status and CYP2B6 metaboliser phenotype: non-pregnant female with normal metabolism (NM), non-pregnant female with intermediate metabolism (IM), non-pregnant female with poor metabolism (PM), and the corresponding pregnant scenarios for each phenotype group. All scenarios used a single oral dose of 600 mg EFV, consistent with standard clinical dosing [27], and PBPK concentration-time profiles were simulated over 72 h following administration. The simulations therefore characterise the disposition of a single EFV dose and do not represent repeated dosing or steady-state exposure. Pregnancy physiology was parameterised to represent the third trimester using the Population Estimates for Age-Related Physiology (PEAR Physiology) module embedded in GastroPlus, which automatically adjusts organ volumes, blood flows, and enzyme expression levels for the selected gestational period; Vmax values for metabolic enzymes were not manually adjusted but were rescaled by the PEAR module from the non-pregnant reference values. The default GastroPlus CYP2B6 parameters for EFV represent an intermediate metaboliser; normal metaboliser (NM) and poor metaboliser (PM) scenarios were generated by scaling CYP2B6 Vmax as described in Table 1. CYP2B6-mediated hepatic intrinsic clearance was parameterised separately for each phenotype group. The CYP2B6*6 allele is associated with reduced enzyme activity, and scaling factors relative to the wild-type were derived from published in vitro-to-in vivo extrapolation data. The NM scenario used a CYP2B6 Vmax of 0.050 mg/s/mg-enzyme; IM scenarios used 70–80% of this value (0.035–0.040 mg/s/mg-enzyme), and PM scenarios used 40–50% (0.020–0.025 mg/s/mg-enzyme), consistent with the approximately twofold lower intrinsic clearance reported for CYP2B6*6/*6 relative to CYP2B6*1/*1 in human liver microsomes [28]. Table 1 summarises these values.

For each of the six scenarios, the Cp-Venous Return profile was analysed from 0 to 72 h after the single 600 mg oral dose. Total plasma concentration was converted from µg/mL to µM using the EFV molecular weight of 315.67 g/mol. The primary free-drug scenario used the fraction unbound implemented in the GastroPlus model, f_u,p_ = 0.0022 (0.22%), to derive the unbound concentration (C_p,u_(t) = C_p,total_(t) × f_u,p_). Because the available export did not contain a directly simulated GastroPlus unbound liver concentration, unbound hepatic concentration was approximated as C_u,liver_(t) ≈ C_p,u_(t) under an explicit free-drug equilibrium assumption. C_u,liver_ is therefore an analytical approximation and must not be interpreted as a directly exported CtU-Liver profile.

### 2.3. In Vitro Pharmacodynamic Data

The pharmacodynamic dataset was obtained from published in vitro experiments by Apostolova and colleagues, who reported the effect of EFV on mitochondrial oxidative stress in Hep3B human hepatic cells using MitoSOX fluorescence as the endpoint [29]. MitoSOX is a cell-permeable fluorescent probe that accumulates selectively in mitochondria and undergoes oxidation by superoxide, providing a surrogate measure of mitochondrial reactive oxygen species (ROS) production. Fluorescence was normalised to the vehicle control, establishing a baseline of 100%.

Concentration-response data were available for four nominal EFV concentrations (0, 10, 25, and 50 µM) measured at 1 h, 6 h, and 24 h, yielding twelve mean observations. These values were extracted from panel A of the figure labelled “Figure 4” in the original publication using WebPlotDigitizer (version 4.6, Ankit Rohatgi), a validated tool for digitising data from published figures. Standard error of the mean (SEM) estimates were also extracted and used as fitting weights in all pharmacodynamic regression steps. The complete extracted dataset is shown in Table 2.

The 24 h timepoint was selected as the primary pharmacodynamic anchor because it produced the most informative concentration-response shape and the most stable model fit across the candidate reference parameterisations. Importantly, the reported EFV concentrations are nominal concentrations added to the in vitro system; unbound extracellular and intracellular EFV concentrations were not available and were therefore not assumed to be equivalent to the derived in vivo unbound concentrations.

### 2.4. Pharmacodynamic Model Fitting and Selection

Oxidative stress was modelled as a direct concentration-effect relationship using a sigmoidal Hill–Emax model. The structural model was:

(1) E(C) = E_0_ + (E_max_ − E_0_) × C*^n^*/(EC_50_*^n^* + C*^n^*)

where E(C) is the predicted MitoSOX fluorescence (%), E_0_ is the baseline fluorescence fixed at 100% based on normalised vehicle control values, Emax is the maximum additional response above baseline (%), C is the EFV concentration (µM), EC_50_ is the concentration producing half-maximal additional response (µM), and *n* is the Hill coefficient controlling the steepness of the sigmoidal relationship. The E_0_ parameter was fixed at 100 because all fluorescence values were normalised to the vehicle control, and the vehicle readings were consistent with this value across all three timepoints (range: 95.65 to 104.34%).

Because the dataset is sparse, with only four concentration levels at 24 h, EC_50_, Emax, and the Hill coefficient could not all be identified reliably from the data. EC_50_ = 15 µM was therefore prespecified as a reference working value for exploratory PBPK-PD mapping rather than selected automatically as a uniquely identified biological constant. Emax and the Hill coefficient were estimated by weighted nonlinear least squares with weights = 1/SEM^2^. Candidate fixed-EC_50_ fits at 10, 15, and 25 µM were additionally evaluated as a technical identifiability audit, comparing weighted residual sum of squares across candidates and checking whether Hill coefficient estimates reached the prespecified upper bound of 10.

The reference Hill–Emax parameterisation was compared with a weighted log-linear model at 24 h using RSS and the Akaike Information Criterion (AIC) as selection criteria. Alternative pharmacodynamic model structures, including a free-baseline model and a Hill-1 model, were evaluated as secondary structural sensitivity analyses. These model comparisons were used to assess the robustness of the exploratory mapping rather than to establish a validated in vivo exposure-response model.

Parameter identifiability was further assessed by profile likelihood for a fixed-EC_50_ model and for a full model in which EC_50_ was estimated freely. The full model used bounded nonlinear least squares (Emax 100–2000, EC_50_ 0.001–500 µM, Hill coefficient 0.1–10). Profile-likelihood results were interpreted as diagnostics of the information content of the four-point 24 h dataset; parameters with profiles that did not cross the 95% threshold were considered poorly or non-identifiable.

### 2.5. PBPK-PD Integration and Primary Pharmacodynamic Endpoints

The fitted 24 h pharmacodynamic surface was applied to the derived unbound exposure profile in each PBPK scenario as an exploratory mapping: E(t) = E_0_ + (E_max_ − E_0_) × C_liver_(t)^n^/(EC_50_^n^ + C_u,liver_(t)^n^), where E_0_ = 100%, EC_50_ = 0.324 µM (the fu,vitro-corrected reference value; Section 2.6), and C_u,liver_(t) ≈ C_p,u_(t). All integrations used the trapezoidal rule implemented with the AUC function from DescTools. This calculation intentionally tests what the **fitted in vitro concentration–response relationship** would predict if applied to the free-drug exposure approximation; it does not establish equivalence between nominal in vitro concentration and in vivo hepatocellular effect-site concentration.

For each scenario, exposure metrics were calculated over the complete 0–72 h PBPK profile. Total plasma Cmax, Tmax, and AUC_0–72_ were obtained from C_p,total_. Corresponding unbound plasma and assumed unbound hepatic Cmax and AUC_0–72_ values were derived using f_u,p_. Exploratory pharmacodynamic summaries included the total area under the effect-time curve (AUCeffect), the baseline-corrected area AUCexcess = ∫0^72 max[E(t) − E_0_,0]dt, maximum effect above E_0_, and time above the reference EC_50_. Because integrating E_0_ = 100% for 72 h contributes a fixed baseline area of 7200%h to AUCeffect, AUCexcess was used to distinguish the drug-associated model signal from this mathematical baseline. Time above EC_50_ was treated as an exploratory exposure-alignment metric, not as time above a validated in vivo biological threshold.

Pregnancy effects were quantified within each CYP2B6 phenotype as pregnant/non-pregnant ratios and percent changes in Cmax and AUC_0–72_. Phenotype effects were quantified relative to the NM scenario within each physiological state. These PBPK scenarios are deterministic simulations rather than population samples; accordingly, fold differences and percent differences were reported without inferential *p*-values. All pharmacodynamic quantities are model-derived values anchored to a surrogate in vitro endpoint and do not represent validated clinical measures of oxidative stress burden in vivo.

### 2.6. Translational Exposure-Gap and Protein-Binding Sensitivity Analysis

The translational distance between predicted unbound exposure and the in vitro concentration range was quantified by comparing assumed C_u,liver_ Cmax with the lowest non-zero nominal in vitro concentration (10 µM) and the reference EC_50_ (15 µM). A literature-anchored f_u,p_ sensitivity analysis used experimentally reported human plasma unbound fractions of 0.20%, 0.22%, and 0.26% from Avery et al. [30] and 0.40%, 0.60%, and 1.50% from Almond et al. [31]. At each value, C_p,u_ = C_p,total_ × f_u,p_ and C_u,liver_ ≈ C_p,u_ were recalculated without introducing an additional hepatic accumulation factor. As a complementary diagnostic, the plasma fraction unbound that would be required, under C_u,liver_ ≈ C_p,u_, for the predicted total plasma Cmax to yield an unbound concentration of 10 µM or 15 µM was calculated as f_u,p_,required = Ctarget/C_p,total_,max. Values greater than 1 indicate that the target cannot be reached even at 100% plasma unbound under this free-drug equilibrium approximation. This exposure-scale diagnostic is independent of Emax and the Hill coefficient.

EFV is extensively bound to serum proteins, and the in vitro concentrations reported by Apostolova et al. were nominal concentrations added to assay medium containing 10% foetal calf serum (FCS). Assuming that EFV binding to FCS is equivalent to its binding in human plasma, the free fraction in the assay medium was derived using the dilution equation of Kalvass and Maurer [32]: fu,vitro = D × f_u,p_/[1 + f_u,p_ × (D − 1)], with a dilution factor D = 10 and f_u,p_ = 0.22%, giving fu,vitro = 2.16%. The nominal concentrations of 10, 25, and 50 µM therefore correspond to free concentrations of 0.22, 0.54, and 1.08 µM, respectively, and the nominal EC_50_ of 15 µM corresponds to an fu,vitro-corrected EC_50_ of 0.324 µM. Because the Hill–Emax equation is invariant to linear rescaling of the concentration axis, Emax (289.9%) and the Hill coefficient (n = 3.05) are preserved unchanged by this correction. The fu,vitro-corrected EC_50_ of 0.324 µM is adopted hereafter as the primary reference value used in the PBPK comparisons that follow, with the nominal EC_50_ (15 µM) retained where explicitly indicated for comparison against the in vitro concentrations as administered. This correction assumes equivalence between EFV–FCS and EFV–human plasma binding, which has not been experimentally validated for EFV in this matrix. This assumption is additional to, and independent of, the free-drug equilibrium approximation already used to derive assumed unbound hepatic exposure from simulated plasma concentrations (C_u,liver_ ≈ C_p,u_; Section 2.2). The fu,vitro-corrected EC_50_ therefore rests on two unvalidated equivalence assumptions rather than one, and the resulting translational-gap estimates should be interpreted accordingly. Notably, the pregnant/non-pregnant AUCeffect,excess ratio was unaffected by this correction (0.316 under both the nominal and the fu,vitro-corrected EC_50_), because this ratio depends algebraically on the relative hepatic exposure between groups rather than on the absolute EC_50_ value in the sub-threshold regime that applies here.

### 2.7. Pharmacodynamic Sensitivity and Uncertainty Analyses

Sensitivity analyses varied the reference EC_50_ across the fu,vitro-corrected range (0.216–0.755 µM, proportionally rescaled from the original nominal grid of 10–35 µM) and jointly varied EC_50_, Hill coefficient, and Emax over prespecified grids. Because the derived unbound concentrations were far below the in vitro range, both total AUCeffect and baseline-corrected AUCexcess ratios were retained to show the effect of the fixed E_0_ contribution. An SEM-based parametric uncertainty analysis generated 1000 perturbed 24 h group-mean datasets around the fitted response using the reported SEM values, refitted Emax and Hill with EC_50_ fixed at the fu,vitro-corrected reference value (0.324 µM), and propagated each converged fit through the PBPK profiles. This procedure represents uncertainty propagation from reported group means and SEMs and is not a nonparametric bootstrap of individual experimental replicates.

A complementary parameter-sampling analysis drew Emax and Hill values from the covariance matrix of the primary fixed-EC_50_ model, with a proportional-uncertainty fallback if the covariance matrix was unavailable, and propagated the sampled parameters through all six scenarios. Alternative pharmacodynamic model structures were also evaluated unconditionally as structural sensitivity analyses.

### 2.8. Statistical Considerations and Model Assumptions

Pharmacodynamic fitting used weighted nonlinear least squares with inverse-SEM^2^ weights. Model comparison used RSS, weighted RSS, and AIC as appropriate, while profile likelihood was used to evaluate parameter identifiability. Missing or non-finite PBPK rows were excluded only when time or total plasma concentration was non-finite; no imputation was performed. The final script contains explicit self-checks confirming that C_p,u_ and assumed C_u,liver_ are identical by construction, that f_u,p_ is applied consistently, that concentrations are non-negative, and that each PBPK profile spans 0–72 h.

The principal translational assumption is C_u,liver_ ≈ C_p,u_ under free-drug equilibrium. This approximation does not account for active hepatic uptake, intracellular accumulation, subcellular partitioning, metabolites, or differences between nominal in vitro concentrations and unbound extracellular or intracellular concentrations. The 24 h timepoint refers to the in vitro pharmacodynamic anchor, whereas 72 h refers to the duration of the PBPK concentration-time simulations and all exposure/effect integrations. These distinct time scales were retained deliberately and should not be conflated.

## 3. Results

### 3.1. Pharmacodynamic Model Fitting

In Table 2, the 24 h Hep3B data showed a steep concentration-dependent MitoSOX response across the nominal 0–50 µM range. Under the prespecified reference EC_50_ of 15 µM, weighted nonlinear least squares estimated Emax = 289.9% and the Hill coefficient *n* = 3.05. The candidate-fit audit demonstrated substantial parameter non-identifiability: the bounded EC_50_ = 25 µM fit had the lowest WRSS but required the Hill coefficient to reach its upper bound of 10. The reference EC_50_ = 15 µM was therefore retained as a working parameterisation rather than interpreted as the uniquely best or biologically identified EC_50_. The 24 h reference Hill–Emax model had an AIC of 48.8 versus 50.5 for the log-linear comparator (Supplementary Figure S1). Accordingly, the fitted PD parameters should not be interpreted as precisely estimated biological constants, but rather as a working parameterisation of the available concentration–response data for the subsequent comparative PBPK–PD analysis.

### 3.2. Baseline Efavirenz Hepatic Exposure and Predicted Oxidative Stress Signal

**Figure 2:**
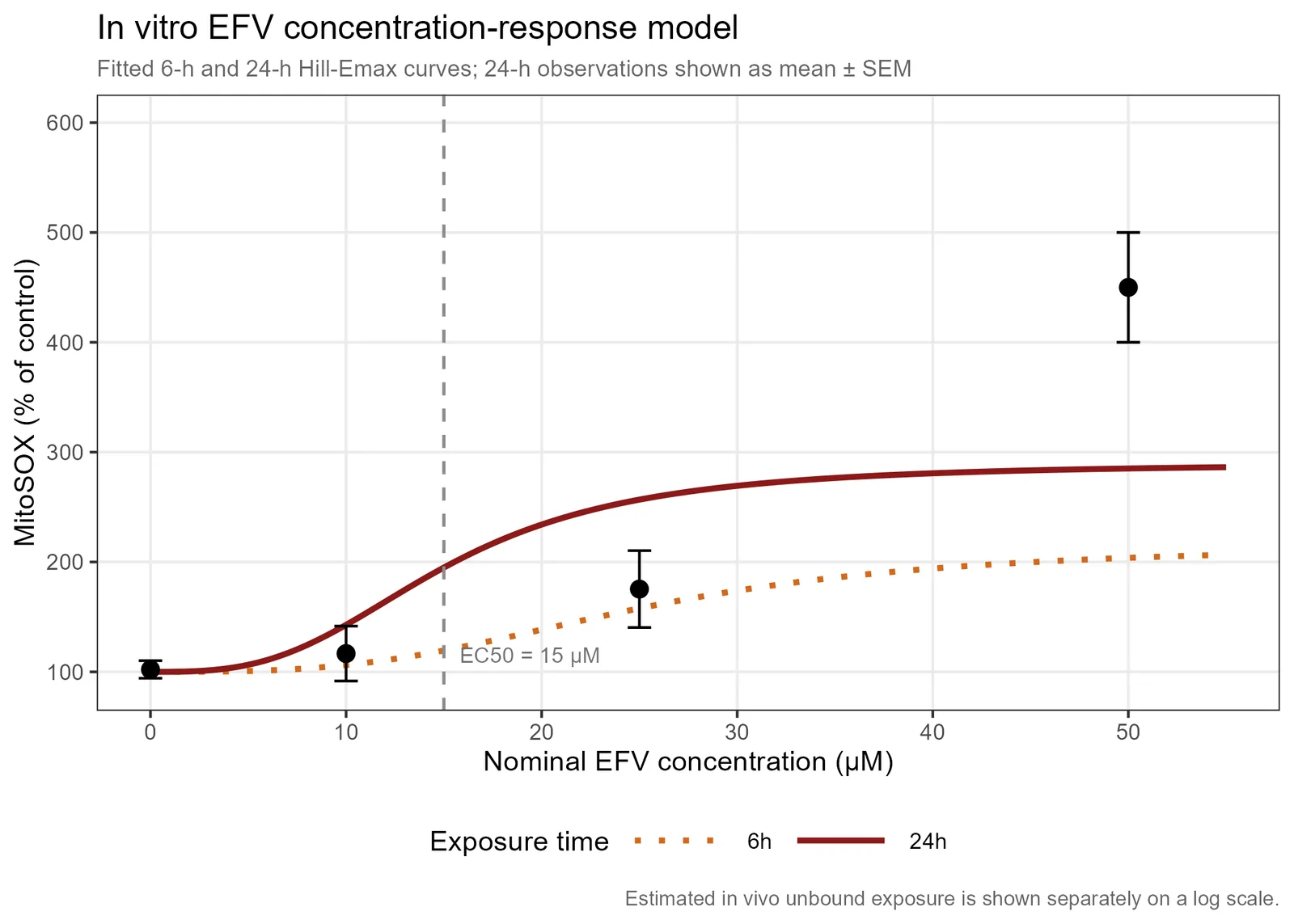
In vitro EFV concentration–response model. Fitted 6 h and 24 h Hill–Emax curves are shown with the 24 h MitoSOX observations (mean ± SEM). The dashed line indicates the reference EC_50_ = 15 µM. Estimated in vivo unbound exposure is presented separately on a logarithmic concentration scale because it lies far below the nominal in vitro concentration range [29].

Figure 2 shows the 6 h and 24 h in vitro concentration–response curves and the observed 24 h means. The reference EC_50_ of 15 µM lies within the nominal experimental range. The PBPK-derived free-drug concentrations are not overlaid on this linear-scale in vitro figure because they are orders of magnitude lower and are instead shown in the dedicated exposure-range comparison.

### 3.3. PBPK Exposure Across Pregnancy and CYP2B6 Phenotypes

All six PBPK profiles covered 0–72 h. In the NM group, pregnancy reduced total plasma Cmax from 9.94 to 7.15 µM (28.1%) and AUC_0–72_ from 200.47 to 118.75 µM·h (40.8%). Pregnancy/non-pregnancy AUC_0–72_ ratios were 0.592, 0.626, and 0.672 for NM, IM, and PM, respectively, while Cmax ratios were 0.719, 0.743, and 0.769. Across phenotypes, PM exposure exceeded NM exposure in both physiological states; total plasma AUC_0–72_ was 28.8% higher in non-pregnant PM than in NM and 46.2% higher in pregnant PM than in NM.

These predicted exposures inherit differential confidence from the underlying PBPK model. In non-pregnant adults, the model previously reproduced observed plasma Cmax within ±25% prediction error at both 400 and 600 mg, and AUC within this range at 400 mg, with AUC underpredicted by approximately 31% at 600 mg. Predictive performance in pregnancy was substantially weaker, with AUC underpredicted by approximately 87% and 48% in two independent third-trimester cohorts [27,33]. The non-pregnant exposure values in Table 3 therefore rest on firmer validation ground than the pregnant values, and the pregnant/non-pregnant ratios reported above should be interpreted with this asymmetry in mind.

Under free-drug approximation, unbound hepatic Cmax values of 0.0219 and 0.0157 µM were assumed in NM non-pregnant and pregnant scenarios, respectively. Across all six scenarios, C_u,liver_ Cmax was assumed to range from 0.0157 to 0.0234 µM, and AUC_0–72_ from 0.261 to 0.568 µM·h. Because the same f_u,p_ multiplier was used in every scenario, relative pregnancy and phenotype ratios are mathematically identical for total plasma, unbound plasma, and assumed unbound liver exposure; this agreement is therefore not independent validation of hepatic exposure.

**Figure 3:**
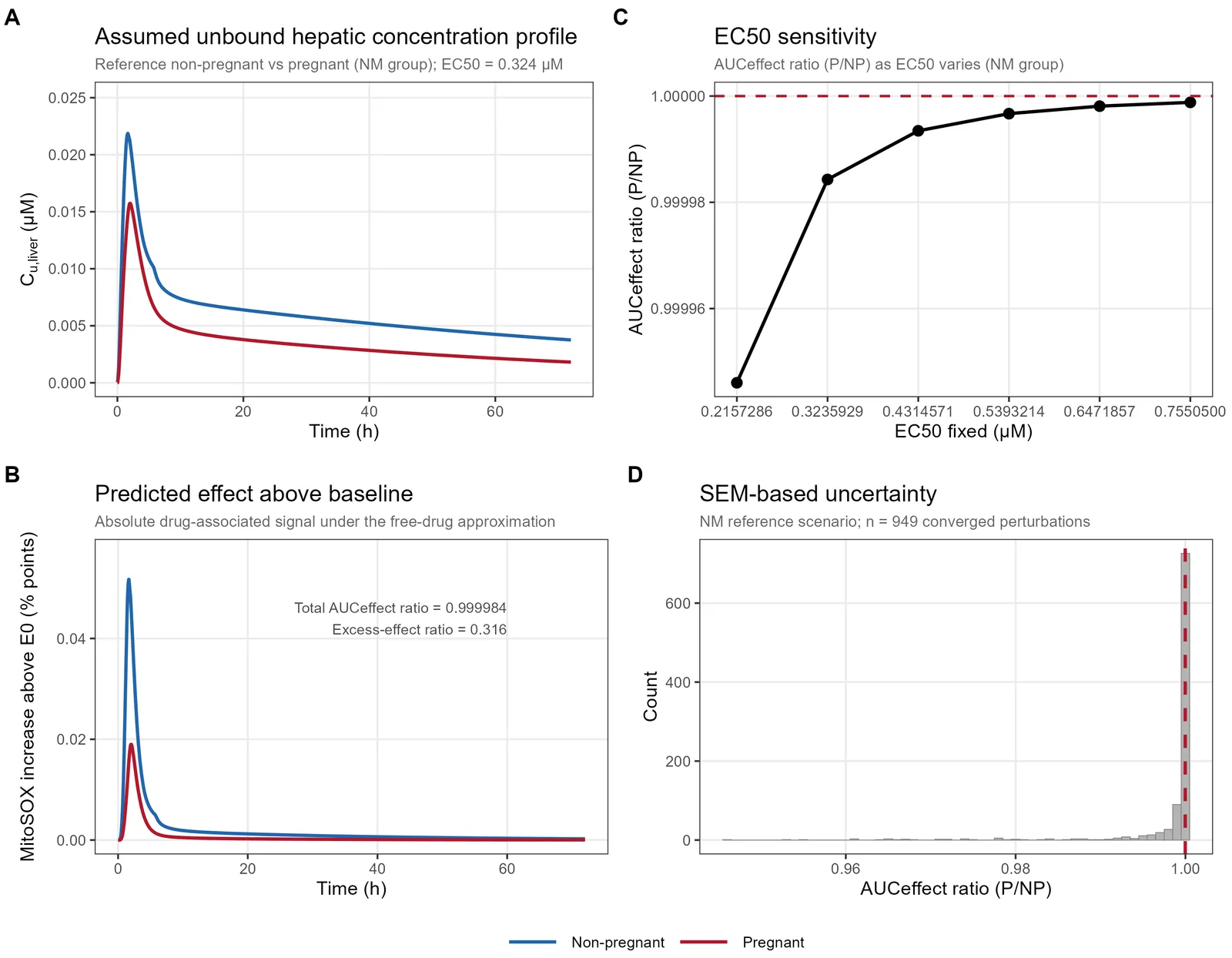
Free-drug reference analysis for the normal-metaboliser group. Panel (**A**): assumed unbound hepatic EFV concentration-time profiles over 0–72 h for non-pregnant (blue) and pregnant (red) scenarios, where C_u,liver_ ≈ C_p,u_ and f_u,p_ = 0.22%. Panel (**B**): predicted MitoSOX increase above E_0_ after applying the 24 h Hill–Emax model, with the fu,vitro-corrected EC_50_ (0.324 µM), to the profiles in panel A. The annotation gives the pregnant/non-pregnant ratios of total AUCeffect and AUCexcess. Panel (**C**): pregnant/non-pregnant ratio of total AUCeffect as a function of the fixed EC_50_. The dashed line marks a ratio of 1. Panel (**D**): distribution of the total AUCeffect ratio from SEM-based parametric perturbation of the 24 h group means. The dashed line marks the median. The extremely small effect-above-baseline scale reflects the large separation between predicted unbound exposure and the nominal in vitro concentration range.

Figure 3 summarises the NM reference comparison under the free-drug approximation. The assumed unbound concentration profiles remain below the fu,vitro-corrected reference EC_50_ (0.324 µM) throughout 72 h, by approximately 15-fold at Cmax in the non-pregnant simulation and 21-fold in pregnancy, so the time above EC_50_ is zero. Direct application of the fitted in vitro Hill–Emax relationship therefore produces effect profiles that are essentially indistinguishable from E_0_. In the NM group, AUCeffect is approximately 7200%h in both states because this quantity is dominated by the fixed 100% baseline integrated over 72 h; the corresponding AUCexcess values are 0.165 and 0.0522%h for non-pregnant and pregnant simulations, respectively. These values demonstrate the numerical consequence of the exposure gap and are not interpreted as quantitative predictions of oxidative stress in vivo.

### 3.4. Translational Exposure Gap and f_u,p_ Sensitivity

At the primary f_u,p_ of 0.22%, the highest assumed unbound Cmax among all six scenarios was 0.0234 µM, making the lowest non-zero in vitro concentration (10 µM) 426.9-fold higher and the reference EC_50_ (15 µM) 640.3-fold higher on the nominal scale; expressed against the fu,vitro-corrected EC_50_ (0.324 µM), the corresponsig gap was approximately 13.8-fold. The exposure gap persisted across all literature-anchored protein-binding assumptions. Even at f_u,p_ = 1.5%, the highest experimentally observed value included in the sensitivity analysis, the maximum assumed unbound Cmax was 0.1597 µM, still 62.6-fold below 10 µM and approximately 2.0-fold below the fu,vitro-corrected EC_50_; no scenario spent any time above EC_50_, although this margin is substantially narrower under the corrected metric than under the nominal in vitro concentration scale.

Across the six scenarios, the plasma fraction unbound required to reach 10 µM or the reference EC_50_ under the free-drug equilibrium approximation was calculated independently of any assumed f_u,p_ value. Even in the most favourable scenario, a minimum of 93.9% plasma unbound would be required to reach the nominal 10 µM concentration, close to but not exceeding the physically possible maximum of 100%. Expressed against the fu,vitro-corrected EC_50_ (0.324 µM), the minimum required plasma fraction unbound was substantially lower still, at 3.04%, well within the physically possible range but still approximately two-fold above the highest literature-reported estimate for efavirenz (1.5%) (Supplementary Figure S7). This confirms that the exposure gap cannot be resolved by uncertainty in the plasma protein binding estimate alone, regardless of which literature value of f_u,p_ is assumed.

### 3.5. Pharmacodynamic Parameter Sensitivity and Uncertainty

Sensitivity analyses were performed to assess the numerical behaviour of the exploratory pharmacodynamic mapping under uncertainty in the 24 h concentration–response model. Across the evaluated EC_50_ values (0.216–0.755 µM, the fu,vitro-corrected sensitivity range), estimated unbound EFV concentrations remained substantially below the corresponding EC50 values. Consequently, integration of the full predicted MitoSOX response over 72 h yielded AUCeffect values of approximately 7200%·h in both non-pregnant and pregnant simulations, reflecting the fixed baseline response (E_0_ = 100%) integrated over the simulation interval rather than a measurable drug-associated effect. Total AUCeffect ratios were therefore numerically indistinguishable from unity and were not considered informative measures of pharmacodynamic equivalence.

Baseline-corrected AUCexcess exposed the small model-derived increment above E_0_ (100%). In the NM reference comparison at the fu,vitro-corrected EC_50_ = 0.324 µM and Hill *n* = 3.05, AUCexcess was 0.165%·h in the non-pregnant simulation and 0.0522%·h in pregnancy. Across the fu,vitro-corrected EC_50_ sensitivity range (0.216–0.755 µM), absolute AUCexcess decreased from 0.568 to 0.0125%·h in the non-pregnant simulation and from 0.179 to 0.00395%·h in pregnancy. Thus, although the relative baseline-corrected response varied with pregnancy-related exposure, its absolute magnitude remained extremely small under the free-drug equilibrium approximation.

Uncertainty propagation from the published MitoSOX data produced 949 valid fits for both total AUCeffect and AUCexcess ratios. The median total AUCeffect ratio was approximately 1.000 (95% interval, 0.9777–1.000), whereas the median AUCexcess ratio was 0.322 (95% interval, 0.0749–0.6399). The difference between these summaries reflects the mathematical contribution of the fixed baseline to total AUCeffect (Supplementary Figure S4). Because the absolute AUCexcess values were several orders of magnitude below 1%·h, the relative AUCexcess ratios should not be interpreted as quantitative estimates of pregnancy-related changes in hepatic oxidative stress in vivo.

Sensitivity to EC_50_, Hill coefficient, Emax, and alternative pharmacodynamic model specifications did not alter the principal interpretation. Under the primary plasma protein-binding assumption, application of the 24 h nominal in vitro concentration–response relationship to the estimated unbound exposure profiles occurs in the extreme low-concentration tail of the fitted response function. This position becomes markedly less extreme toward the upper end of the literature-reported protein-binding range (Section 4.1) (Supplementary Figures S5 and S6). Across the EC_50_ × Hill grid (90 combinations, EC_50_ = 0.216–0.755 µM, Hill *n* = 2.0–4.0), the mean AUCexcess ratio was 0.357 (SD 0.062; range 0.253–0.485); across the Emax × Hill grid (195 combinations, Emax = 200–500), the mean AUCexcess ratio was 0.357 (SD 0.062; range 0.254–0.485). In both grids, 100% of parameter combinations produced an AUCexcess ratio below unity, and the ratio was invariant to EC_50_ and Emax at fixed Hill, reflecting algebraic cancellation of both parameters in the pregnant/non-pregnant ratio (Supplementary Figures S5 and S6). Accordingly, these analyses characterise uncertainty in the exploratory pharmacodynamic extrapolation but cannot resolve the absence of a directly matched effect-site exposure metric between the in vitro and PBPK systems.

### 3.6. Alternative Pharmacodynamic Model Specifications

Alternative pharmacodynamic model specifications were evaluated to determine whether the numerical behaviour of the exploratory mapping was dependent on the selected Hill–Emax structure. A model in which E_0_ was estimated freely and a simplified Emax model with the Hill coefficient fixed at 1 were fitted to the 24 h MitoSOX data and propagated through the same derived unbound exposure profiles.

Under the free-E_0_ specification, the fitted baseline was approximately 102.6%, and the resulting total effect-time integrals were dominated by this baseline contribution. Similarly, the Hill-1 model generated total effect-time integrals close to their corresponding baseline integral, despite producing a larger incremental response than the reference Hill–Emax parameterisation. Thus, near-unity ratios of total AUCeffect across alternative model structures primarily reflected integration of the baseline response over 72 h and were not interpreted as evidence of pharmacodynamic equivalence between pregnant and non-pregnant scenarios.

Across the alternative specifications, the absolute model-derived response above baseline remained small relative to the integrated baseline under the free-drug exposure approximation. Structural sensitivity therefore did not alter the principal interpretation that the pharmacodynamic calculations represent extrapolation in the low-concentration region of models fitted to nominal in vitro concentrations substantially above the estimated unbound in vivo exposure. Parameter-sampling under joint Emax, EC_50_, and Hill uncertainty produced median AUCexcess ratios of 0.312 (NM), 0.338 (IM), 0376 (PM) (Supplementary Figure S4). For the free-E_0_ specification, the fitted baseline was E_0_ = 102.6%, with Emax constrained at its upper bound of 2000%, EC_50_ = 92.7 µM, and Hill coefficient *n* = 2.43 (RSS = 40.5). For the Hill-1 specification, Emax was likewise constrained at its upper bound of 2000%, with EC_50_ = 335.8 µM (RSS = 15,407.7). The boundary-constrained Emax and implausible EC_50_ values in both alternative specifications further illustrate the weak identifiability of the underlying four-point dataset.

## 4. Discussion

This study investigated whether a published concentration-dependent mitochondrial superoxide response to efavirenz (EFV) in hepatic cells could be quantitatively related to human exposure using an integrated PBPK–PD framework, and whether this relationship was affected by physiologically and genetically driven differences in EFV pharmacokinetics. The principal finding was that the apparent translational relationship depended critically on the concentration metric used to connect the PBPK and in vitro systems. Total hepatic EFV concentrations predicted by the PBPK model can occupy the same numerical concentration range as the nominal concentrations associated with MitoSOX responses in Hep3B cells. However, total tissue concentrations and nominal culture-medium concentrations do not demonstrate equivalent effect-site exposures. When the analysis was instead expressed using an unbound-drug approximation, estimated unbound concentrations remained substantially below the nominal in vitro concentration range across pregnancy states and CYP2B6 phenotypes. Consequently, the available data do not support a unique quantitative mapping between clinically predicted EFV exposure and the published mitochondrial superoxide concentration–response relationship.

### 4.1. The Exposure-Metric Problem: Total Versus Unbound EFV Concentration

This distinction is particularly important for EFV because of its extensive protein binding. The original numerical correspondence between PBPK-predicted total liver concentrations and the 10–50 µM nominal EFV concentrations used in the Hep3B experiments may suggest that the experimentally observed mitochondrial response occurs within a clinically relevant hepatic concentration range. Such a comparison, however, implicitly treats two different exposure metrics as quantitatively interchangeable. Total liver tissue concentration includes both bound and unbound drug and may additionally reflect heterogeneous distribution among tissue compartments, whereas nominal in vitro concentration represents the amount of drug added to the culture medium and does not establish the concentration available to the intracellular or mitochondrial effect site. Numerical overlap between these quantities therefore cannot, by itself, establish pharmacodynamic exposure matching.

The free-drug analysis provides a complementary, although likewise assumption-dependent, perspective. Under the free-drug equilibrium approximation, unbound hepatic exposure was represented by unbound plasma EFV concentration (C_u,liver_ ≈ C_p,u_) [30]. Using the reference plasma fraction unbound of 0.22%, the resulting estimated unbound concentrations were several orders of magnitude below the nominal concentrations used in the Hep3B experiments and below the fu,vitro-corrected EC_50_ (0.324 µM) used as the primary reference value in the exploratory pharmacodynamic model. Importantly, this separation was not explained simply by selection of a single protein-binding value. Sensitivity analysis across literature-reported values up to 1.5% increased the estimated unbound exposure, but even at this upper value, the maximum estimated concentration remained approximately 0.16 µM, more than 60-fold below the lowest non-zero nominal in vitro concentration of 10 µM and approximately 2-fold below the fu,vitro-corrected EC_50_, a substantially narrower margin than suggested by the nominal in vitro concentration scale (see Section 4.3 for discussion of the additional uncertainty contributed by PBPK model validation). Thus, within the assumptions of the free-drug framework, plausible uncertainty in plasma protein binding alone was insufficient to reconcile the predicted in vivo exposure with the nominal concentration range associated with the experimental MitoSOX response.

The magnitude of this exposure gap should not, however, be interpreted as evidence that EFV cannot produce mitochondrial oxidative effects in vivo. The free-drug comparison does not constitute a validated free-to-free in vitro-to-in vivo extrapolation because the corresponding unbound EFV concentration in the Hep3B experiments was not reported. Moreover, extracellular unbound concentration may not equal intracellular or mitochondrial exposure. Cellular uptake, membrane partitioning, intracellular binding, transporter activity, subcellular accumulation, and metabolism may generate substantial differences between nominal medium concentration and the concentration experienced at the mitochondrial effect site. The present data therefore define neither the unbound in vitro EC_50_ nor the intracellular concentration associated with the observed MitoSOX response. The free-drug analysis is consequently best interpreted as a sensitivity framework demonstrating how strongly the apparent translational relationship changes when exposure is expressed on an unbound rather than total-tissue basis, rather than as proof of an absolute concentration threshold for oxidative stress in vivo.

### 4.2. Comparison with Prior Pharmacodynamic and Clinical Evidence

The biological observations underlying the pharmacodynamic component nevertheless remain relevant. Apostolova and colleagues reported a concentration-dependent increase in mitochondrial superoxide production in Hep3B cells exposed to EFV, together with alterations in mitochondrial membrane potential and glutathione homeostasis. These findings provide experimental evidence that EFV can perturb mitochondrial redox biology under the tested conditions. The present analysis does not challenge that observation; rather, it addresses the separate question of whether the concentrations producing that response can be quantitatively related to clinically relevant human exposure. This distinction is important because the biological plausibility of a mechanism and the quantitative demonstration that the corresponding effect-site exposure is achieved in vivo are not equivalent forms of evidence.

The 10–50 µM range used by Apostolova et al. is consistent with concentrations broadly described as ‘clinically relevant’ across related in vitro studies from the same research group, including comparative mitochondrial toxicity assessments of efavirenz against newer antiretrovirals [34]. Predicted unbound hepatic EFV exposure remained well below this range across all six scenarios (Section 3.3), a separation unlikely to be explained by PBPK prediction error alone given its typical magnitude (Section 4.3).

Clinical observations provide additional biological context but do not resolve this question. Studies in people with HIV receiving antiretroviral therapy have reported changes in systemic oxidative stress markers, including lipid peroxidation products, antioxidant indices, and markers of oxidative DNA damage [12,35]. Such findings support the plausibility of altered redox homeostasis during antiretroviral treatment but cannot be directly mapped onto the present model. These biomarkers are systemic, may reflect multiple drugs and disease-related processes, and do not quantify hepatocellular or mitochondrial EFV exposure. Conversely, the MitoSOX endpoint used here represents a drug-specific experimental response in a hepatic cell line and has not been validated as a clinical biomarker of EFV-associated liver injury. The PBPK–PD framework should therefore not be interpreted as predicting clinical oxidative stress burden or hepatotoxicity.

### 4.3. Pregnancy- and Phenotype-Related Exposure Differences

Pregnancy and CYP2B6 phenotype were included in the framework as clinically relevant sources of variation in EFV exposure rather than as novel pharmacokinetic findings. Their value in the present analysis is that they provide physiologically and genetically distinct exposure scenarios with which to test the stability of the in vitro-to-in vivo relationship. Although these factors altered the magnitude and time course of PBPK-predicted EFV exposure, they did not resolve the fundamental exposure-metric uncertainty. Under the free-drug approximation, the estimated concentrations remained well below the nominal in vitro concentration range across all six scenarios. Thus, the principal translation limitation is not whether pregnancy or CYP2B6 phenotype produces a modest upward or downward shift in EFV exposure, but whether the PBPK-derived and experimental concentrations represent comparable measures of drug availability at the relevant biological effect site.

The PBPK component nevertheless contributes uncertainty to this comparison. The model was based on a previously developed EFV framework and reproduced established genotype- and pregnancy-associated pharmacokinetic behaviour, but its previous evaluation in pregnancy showed greater prediction error than in non-pregnant adults. The simulated hepatic profiles should therefore be regarded as scenario-level exposure estimates rather than exact predictions of individual hepatic concentrations. Because the present analysis used deterministic profiles for each CYP2B6 group, within-group pharmacokinetic variability was also not represented. These limitations affect the precision of the predicted exposure distributions, although they are unlikely on their own to account for the large separation observed between estimated unbound concentrations and the nominal in vitro concentration range under the free-drug approximation.

### 4.4. Consequences for the Exploratory Pharmacodynamic Mapping

The exploratory Hill–Emax mapping further illustrates the consequences of this exposure separation. The 24 h MitoSOX dataset contained only four nominal concentration levels, and the pharmacodynamic parameters were consequently weakly identified: Emax was boundary-constrained, the Hill coefficient was uncertain, and EC_50_ could not be robustly estimated without prespecification of candidate values. The 15 µM EC_50_ should therefore be regarded as a reference working parameterisation rather than a uniquely identified biological constant. More importantly, when the fitted concentration–response relationship was applied to the derived unbound exposure profiles, the model operated far below the experimentally sampled non-zero concentration range. Predictions in this region are determined primarily by extrapolation of the mathematical form of the concentration–response function rather than by direct experimental observations. The fu,vitro-corrected value (0.324 µM), obtained by applying the Kalvass and Maurer dilution correction for efavirenz protein binding in the 10% foetal calf serum assay medium (Section 2.6), was adopted as the primary reference EC_50_ for the PBPK-linked exposure comparisons; because the Hill–Emax equation is invariant to linear rescaling of the concentration axis, this correction changes the numerical EC_50_ value but not the underlying parameter identifiability limitations described above.

This issue also explains the behaviour of the integrated pharmacodynamic endpoints. Integration of the full predicted response over 72 h yields a total AUCeffect of approximately 7200%·h even in the absence of a meaningful drug-associated increment because the baseline response (E_0_ = 100%) is itself integrated over the complete simulation interval. Near-unity pregnant/non-pregnant AUCeffect ratios are therefore largely a mathematical consequence of the baseline contribution and should not be interpreted as evidence of pharmacodynamic equivalence or saturation. Baseline-corrected AUCexcess provides a more transparent description of the model-derived increment, but its absolute values under the free-drug approximation are extremely small, and its relative ratios remain dependent on extrapolation of the poorly constrained low-concentration region of the fitted model. Sensitivity to EC_50_, Hill coefficient, Emax, uncertainty in the experimental observations, and alternative pharmacodynamic structures therefore characterises the numerical behaviour of the exploratory mapping but cannot overcome the absence of a validated common effect-site exposure metric.

### 4.5. Novelty and Translational Significance

The main contribution of the present framework is therefore not a quantitative prediction of hepatic oxidative stress burden in pregnancy, but the explicit evaluation of the exposure assumptions required to connect a cellular safety endpoint with human PBPK predictions. The analysis demonstrates that apparently straightforward integration of PBPK-derived tissue concentrations with an in vitro concentration–response relationship can lead to markedly different interpretations depending on whether total tissue, nominal medium, or estimated unbound exposure is used. This issue is particularly relevant for highly protein-bound compounds such as EFV and is not resolved by increasing the complexity of the downstream pharmacodynamic model. In this setting, uncertainty in exposure matching precedes, and is more consequential than, uncertainty in the Hill–Emax parameters themselves.

From a translational modelling perspective, this distinction provides a practical criterion for interpreting future PBPK–PD safety analyses. Before a concentration–response relationship derived in vitro is projected onto an in vivo tissue profile, the concentration metrics on both sides of the bridge should be defined explicitly and their comparability justified. For the present EFV dataset, measurement of unbound drug in the culture medium, together with intracellular and ideally mitochondrial EFV concentrations, would substantially strengthen this bridge. Such measurements would allow the experimental response to be expressed against an effect-site-relevant exposure metric and would determine whether the apparent gap observed under the present free-drug approximation reflects a genuine difference in exposure or incomplete in vitro dosimetry.

### 4.6. Limitations and Future Directions

Several limitations should be considered when interpreting these findings. First, MitoSOX fluorescence is a surrogate measure of mitochondrial superoxide production and has not been validated as a quantitative biomarker of EFV-associated hepatocellular injury or clinical toxicity. Second, the pharmacodynamic dataset is sparse and was obtained in Hep3B cells at nominal concentrations, without corresponding measurements of unbound extracellular, intracellular, or mitochondrial EFV. The resulting Hill–Emax model is therefore an exploratory representation of the observed 24 h concentration–response pattern rather than a fully identified pharmacodynamic model. Third, the approximation assumes free-drug equilibrium and does not account explicitly for active transport, intracellular binding, lysosomal or membrane partitioning, subcellular accumulation, or locally generated metabolites. It should consequently be interpreted as a sensitivity scenario rather than a measured hepatic effect-site concentration.

The PBPK simulations represent deterministic scenario-level profiles and do not capture within-phenotype interindividual variability. In addition, uncertainty in the pregnant PBPK predictions was not formally propagated through the full translational analysis. The 24 h in vitro response was applied as a concentration-dependent mapping across the 72 h PBPK profiles, which assumes that the fitted 24 h relationship can be used instantaneously over time and does not incorporate delay, adaptation, cumulative mitochondrial injury, recovery, or turnover of the oxidative stress response. These assumptions limit the interpretation of the time-integrated pharmacodynamic outputs and reinforce their role as exploratory model quantities rather than predictions of an in vivo biological time course. In addition, the 24 h MitoSOX values used to anchor the pharmacodynamic model were digitised from a published figure (Apostolova et al., 2010) using WebPlotDigitizer, an approach with established reliability for recovering quantitative data from graphical sources [36,37]. Nonetheless, the digitised values should be regarded as approximate reconstructions of the original experimental measurements rather than raw data, and this adds a further source of uncertainty to the already sparse four-point concentration-response dataset.

The most informative next step would be the experimental characterisation of EFV dosimetry in the in vitro system used to define the mitochondrial response. Measurement of unbound EFV in culture medium over the exposure period, together with intracellular and subcellular concentrations where feasible, would allow the concentration–response relationship to be reconstructed on a mechanistically relevant exposure basis. Additional concentrations below 10 µM would also be particularly valuable because the current dataset provides no direct information in the concentration region approached by the free-drug PBPK estimates. These data could then support a more defensible quantitative in vitro-to-in-vivo extrapolation and determine whether a dynamic PBPK–PD model of mitochondrial oxidative stress is warranted. Population PBPK simulations and clinical oxidative stress biomarkers could subsequently extend such a framework, but their interpretation would remain limited until the exposure bridge itself is experimentally characterised.

Overall, the present analysis supports the biological plausibility of EFV-associated mitochondrial oxidative stress while placing important limits on its quantitative translation from the available Hep3B data to human hepatic exposure. The key uncertainty is not whether EFV pharmacokinetics vary with pregnancy or CYP2B6 phenotype, nor whether different Hill–Emax parameterisations can generate different modelled effects, but whether the concentration driving the experimental mitochondrial response can be quantitatively matched to an appropriate in vivo hepatic effect-site exposure. Until that relationship is established, PBPK–PD projections of this endpoint should be interpreted as hypothesis-generating rather than as quantitative predictions of oxidative stress burden.

## 5. Conclusions

This study developed and applied a PBPK-PD framework integrating simulated efavirenz exposure across pregnancy status and CYP2B6 phenotype with an in vitro Hill–Emax pharmacodynamic model anchored to Hep3B MitoSOX fluorescence data. Pregnancy and CYP2B6 phenotype altered simulated EFV exposure in the expected direction, but quantitative translation of this exposure to the in vitro oxidative stress response depended critically on the concentration metric used to connect the two systems. Total hepatic EFV concentrations numerically overlapped the nominal in vitro concentration range, whereas estimated unbound hepatic concentrations, derived under an explicit free-drug equilibrium approximation, remained at least 62-fold below the lowest non-zero in vitro concentration across all six scenarios and across the full range of literature-reported plasma protein binding. Expressed against the fu,vitro-corrected in vitro EC_50_, which accounts for efavirenz protein binding in the assay medium, this margin narrowed to approximately 15- to 21-fold at the primary plasma protein-binding value and to approximately 2-fold at the highest literature-reported value, indicating that the translational gap is substantially narrower, though not closed, once the in vitro exposure metric is itself corrected for protein binding. Because unbound EFV was not reported in the in vitro system and intracellular unbound hepatic EFV could not be obtained from the PBPK outputs, neither comparison establishes a validated effect-site exposure match, and the resulting AUCeffect calculations should be interpreted as an exploratory QIVIVE mapping rather than a quantitative prediction of in vivo hepatic oxidative stress burden. These results are model-based and constrained by sparse in vitro data, non-identifiable pharmacodynamic parameters, and assumptions inherent in vitro-to-in vivo extrapolation. They do not constitute clinical evidence and should not be translated to patient-level safety guidance without direct measurement of unbound EFV concentrations in the in vitro system and clinical validation of the exposure-response relationship. The framework itself, and the exposure-matching criterion it makes explicit, represents a replicable approach to evaluating whether pregnancy- and genotype-driven pharmacokinetic differences are pharmacodynamically consequential, applicable to other antiretroviral agents for which in vitro concentration-response data exist.

## Figures and Tables

**Table 1:** CYP2B6 phenotype groups and hepatic intrinsic clearance parameters used in PBPK simulations. Scaling factors are derived from published in vitro data on CYP2B6*6 allele activity. IM: intermediate metaboliser; NM: normal metaboliser; PM: poor metaboliser.

CYP2B6 Phenotype	Genotype (Representative)	Vmax (mg/s/mg-Enzyme)	Scaling Relative to NM
Normal metaboliser (NM)	CYP2B6*1/*1	0.050	Reference
Intermediate metaboliser (IM)	CYP2B6*1/*6	0.035–0.040	70–80% of NM
Poor metaboliser (PM)	CYP2B6*6/*6	0.020–0.025	40–50% of NM

**Table 2:** MitoSOX fluorescence (% of vehicle control) in Hep3B cells exposed to efavirenz at four concentrations and three time points. Data digitised from panel A of the figure labelled “Figure 4” of Apostolova et al. [29]. SEM values were estimated from the error bars in the original figure and used as regression weights. Significance symbols: * *p* < 0.05, *** *p* < 0.001 vs. vehicle at corresponding time, as reported in the original publication.

EFV Concentration (µM)	MitoSOX 1 h (%)	MitoSOX 6 h (%)	MitoSOX 24 h (%)	SEM (Pooled Estimate)
0 (vehicle)	95.65	104.34	102.17	8
10	97.10	111.60	116.66	10–25
25	105.80	140.60 *	175.36 *	15–35
50	133.33 ***	219.56 ***	450.00 ***	25–50

**Table 3:** PBPK-predicted total plasma and derived unbound EFV exposure over 0–72 h for all six scenarios. C_p,total_ values are direct GastroPlus Cp-Venous Return outputs converted to µM. C_p,u_ = C_p,total_ × f_u,p_ using f_u,p_ = 0.0022, and C_u,liver_ ≈ C_p,u_ is an explicit free-drug equilibrium approximation rather than a directly exported GastroPlus liver concentration. Ratios compare pregnant with non-pregnant simulations within each CYP2B6 phenotype.

Group	Status	C_p,total_ Cmax (µM)	C_p,total_ AUC_0–72_ (µM·h)	C_u,liver_ Cmax (µM)	C_u,liver_ AUC_0–72_ (µM·h)	Cmax Ratio (P/NP)	AUC Ratio (P/NP)	Time Above EC_50_ (h)
NM	Non-preg	9.939	200.474	0.02187	0.4410	0.719	0.592	0
NM	Pregnant	7.151	118.748	0.01573	0.2612	0.719	0.592	0
IM	Non-preg	10.282	226.270	0.02262	0.4978	0.743	0.626	0
IM	Pregnant	7.635	141.597	0.01680	0.3115	0.743	0.626	0
PM	Non-preg	10.648	258.249	0.02343	0.5681	0.769	0.672	0
PM	Pregnant	8.186	173.607	0.01801	0.3819	0.769	0.672	0

## Data Availability

The original contributions presented in this study are included in the article/Supplementary Material. Further inquiries can be directed to the corresponding author.

## Supplementary Materials

The following supporting information can be downloaded at https://www.mdpi.com/article/10.3390/pharmaceutics18101253/s1: Supplementary Note S1: Analytical code description; Figure S1: Comparison of pharmacodynamic model fits to the 24 h in vitro MitoSOX concentration-response data; Figure S2: Profile likelihood functions for the unconstrained Hill–Emax model parameters; Figure S3: Summary of pharmacokinetic and pharmacodynamic ratio metrics in the normal metaboliser reference scenario; Figure S4: Parametric parameter-sampling AUCeffect ratio distributions under joint uncertainty in Emax, EC_50_, and Hill coefficient; Figure S5: Two-dimensional EC_50_ × Hill coefficient sensitivity surface for modelled AUCeffect ratios; Figure S6: Two-dimensional Emax × Hill coefficient sensitivity surface for modelled AUCeffect ratios; Figure S7: Plasma fraction unbound required, under the free-drug equilibrium approximation, to reach the lowest non-zero in vitro concentration (10 µM) or the fu,vitro-corrected reference EC_50_ (0.324 µM) across all six scenarios; Table S1: Physicochemical and biopharmaceutical parameters for the efavirenz PBPK model used in GastroPlus v.9.8.3.

## Abbreviations

The following abbreviations are used in this manuscript:

PBPK-PD	Physiologically based pharmacokinetic–pharmacodynamic
CYP2B6	Cytochrome P450 2B6
AUCeffect	Area under the pharmacodynamic effect–time curve
EC_50_	Half-maximal effective concentration
Emax	Maximum additional response above baseline
ART	Antiretroviral therapy
HIV	Human immunodeficiency virus
NRTI	Nucleoside reverse transcriptase inhibitor
ROS	Reactive oxygen species
EFV	Efavirenz
PBPK	Physiologically based pharmacokinetic
CYP3A4	Cytochrome P450 3A4
NM	Normal metaboliser
IM	Intermediate metaboliser
PM	Poor metaboliser
PEAR	Population Estimates for Age-Related Physiology
Vmax	Maximum enzymatic velocity
Km	Michaelis constant
Cliver	Simulated hepatic unbound efavirenz concentration
SEM	Standard error of the mean
E_0_	Baseline fluorescence
NLS	Nonlinear least squares
RSS	Residual sum of squares
AIC	Akaike information criterion
Cmax	Peak hepatic concentration
AUCliver	Hepatic area under the concentration–time curve
Tmax	Time to peak concentration
