# Supplementary material for: Integrating PBPK Simulation and In Vitro Pharmacodynamics to Model Efavirenz-Associated Hepatic Oxidative Stress in Pregnancy
Source: Pharmaceutics. 2026 Oct 2;18(10):1253. doi: 10.3390/pharmaceutics18101253 (PMC13708575; doi:10.3390/pharmaceutics18101253)
Supplement: Supplementary file 1 [file pharmaceutics-18-01253-s001.zip › pharmaceutics-4488442-supplementary.pdf]

Supplementary Material

Integrating PBPK Simulation and In Vitro Pharmacodynamics to ModelEfavirenz-Associated Hepatic Oxidative Stress in Pregnancy

SUPPLEMENTARY NOTE S1. ANALYTICAL CODE DESCRIPTION

Software and environment

All analyses were performed in R (version 4.5.3; R Foundation for Statistical Computing, Vienna, Austria). The following packages were used: ggplot2 for figure generation; DescTools for trapezoidal area under the curve computation; patchwork for multi-panel figure assembly; dplyr and tidyr for data manipulation; readxl for importing GastroPlus Excel output; MASS (accessed via explicit namespace qualification, MASS::mvrnorm(), to avoid masking dplyr::select()) for covariance-based parametric sampling . A random seed of 42 was set at the start of the analysis script to ensure full reproducibility of all stochastic procedures including the parametric bootstrap.

GastroPlus v.9.8.3 (Simulation Plus Inc., Lancaster, CA, USA) was used for PBPK simulation. Concentration-time profiles were exported as Excel files and imported into R for pharmacodynamic processing.

Code availability. The full annotated R script is available from the corresponding author upon reasonable request. The script is structured in nine sequential blocks (Blocos 1-9) covering in vitro data preparation, PBPK import, reference analysis, pregnancy comparison, IM/PM scenario analysis, dose comparison, profile likelihood, multivariate robustness analysis, and alternative model specifications.

SUPPLEMENTARY TABLE S1

Supplementary Table S1. Physicochemical and biopharmaceutical parameters for the efavirenz PBPK model used in GastroPlus v.9.8.3. Values represent a mix of experimental, predicted, and fitted values as implemented in the GastroPlus compound library for efavirenz. The CYP2B6 $V_{max,PBPK}$ value shown (0.050 mg/s/mg-enzyme) corresponds to the normal metaboliser (NM; CYP2B6*1/*1) reference scenario; intermediate and poor metaboliser values were derived by applying the scaling factors described in Table 1 of the main text. Pregnancy-related enzyme induction parameters (E_{max} and EC_{50}) were applied automatically by the PEAR Physiology module for the pregnant scenarios. Source: Simulation Plus Inc.,

GastroPlus compound library for efavirenz [Darling I. MIDD for Efavirenz. Simulation Plus Inc.; available from: https://www.simulations-plus.com/wp-content/uploads/MIDD-Inger-Darling-efavirenz.pdf] and the prior PBPK modelling study by this group [reference 26 in main text].

**Efavirenz property**	**Value**
logP (at pH = 7.4)	4.6
Aqueous solubility at pH 6.99	9.0 mg/L
pKa (acid)	10.2
Permeability: Caco-2 Papp	0.25 cm/s × 10^{-5}
Permeability: Human Peff	1.07 cm/s × 10^{-4} [b]
Fraction unbound in plasma (human)	0.22%
Blood/plasma concentration ratio (human)	0.74
Mean particle radius	5 µm
CYP3A4 Km	3.137 µg/mL
CYP3A4 Vmax,PBPK	0.000562 mg/s/mg-enzyme
CYP3A4 Vmax,GUT	0.2 mg/s
CYP2B6 Km	3.137 µg/mL
CYP2B6 Vmax,PBPK (NM reference)	0.050 mg/s/mg-enzyme
CYP3A4 Emax (pregnancy induction)	9.9
CYP3A4 EC50 (pregnancy induction)	1.0 µM
CYP2B6 Emax (pregnancy induction)	5.76
CYP2B6 EC50 (pregnancy induction)	0.82 µM

[a] Values represent a mix of experimental, predicted, and fitted inputs. Experimental values were obtained from published literature; predicted values were generated by GastroPlus using structure-based estimation methods; fitted values were optimised against pharmacokinetic data. For individual source attribution, see the Simulation Plus MIDD technical report cited above.

[b] Caco-2 Papp was converted to human Peff using the built-in ABSCa conversion implemented in GastroPlus.

CYP2B6 Km: the same Km value as CYP3A4 (3.137 µg/mL) is listed in the source material; this is the value as implemented in the GastroPlus model and was not altered in the present analysis.

CYP3A4 and CYP2B6 Emax and EC50 values describe the pregnancy-related enzyme induction parameterisation as implemented in the PEAR Physiology module. They define the concentration-dependence of gestational hormone-driven enzyme upregulation, not the pharmacodynamic concentration-response relationship used in the MitoSOX analysis.

SUPPLEMENTARY FIGURE S1

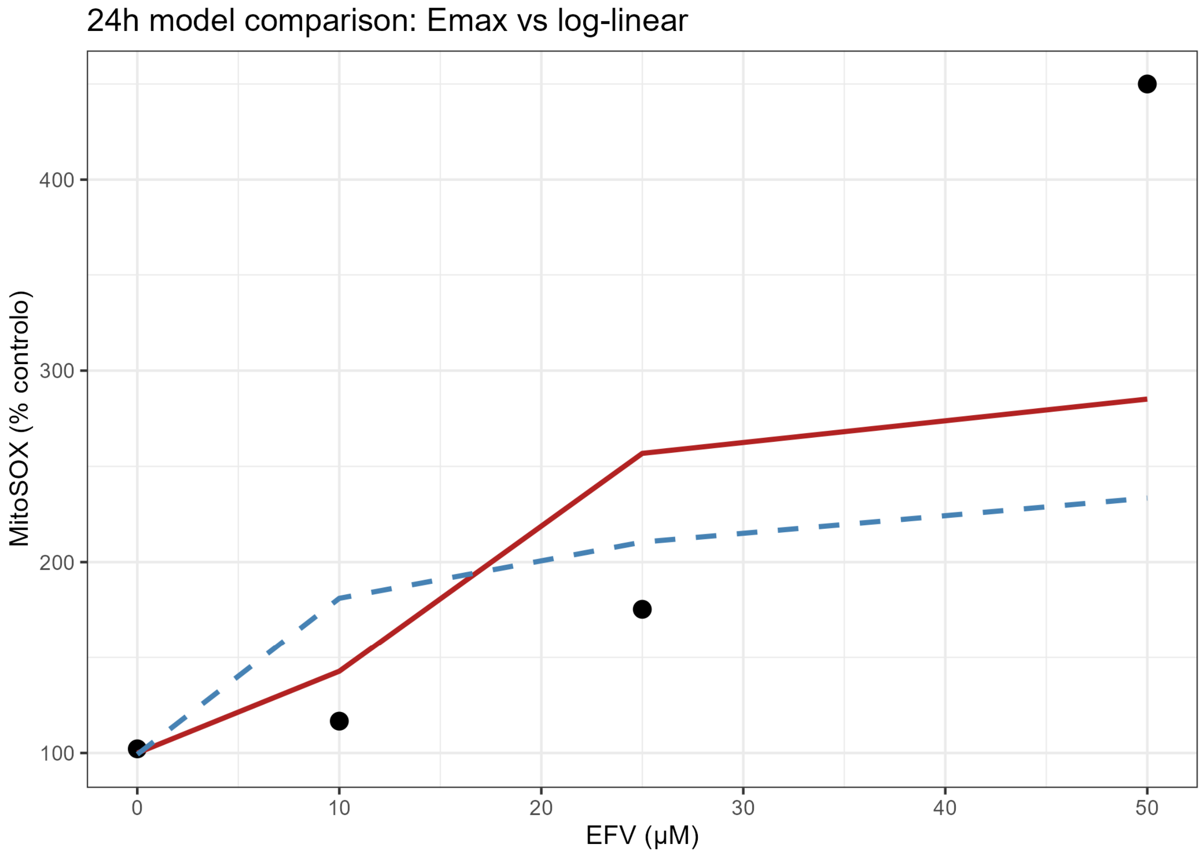

Supplementary Figure S1. Comparison of pharmacodynamic model fits to the 24-hour in vitro MitoSOX concentration-response data. The solid red curve shows the fitted Hill-Emax model (EC50principal = 15 µM; Hill coefficient n = 3.05; Emax boundary-constrained at the upper estimation limit). The dashed blue curve shows the fitted log-linear alternative. Points represent observed in vitro data (mean, digitised from Apostolova et al., 2010). The Hill-Emax model was preferred based on both RSS (34,497 vs 52,289) and AIC (48.8 vs 50.5). Neither model captures the observed value at 50 µM, 24 hours (450% of control), which lies above the modelled plateau.

SUPPLEMENTARY FIGURE S2

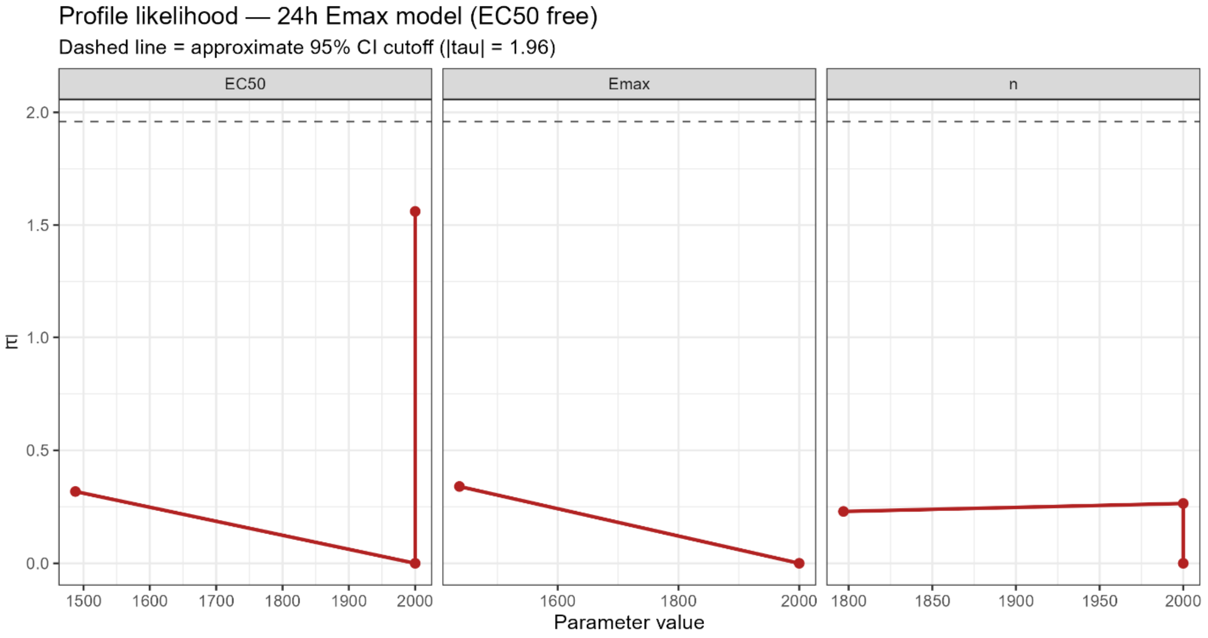

Supplementary Figure S2. Profile likelihood functions for the three pharmacodynamic model parameters (Emax, EC50, Hill coefficient n) from the unconstrained Hill-Emax model in which all three parameters were estimated freely. The horizontal dashed line marks the chi-squared threshold $|\tau| = 1.96$ defining the 95% confidence interval boundary. Vertical dashed lines indicate point estimates (EC50 = 93.97 µM; Emax = 2,000, boundary; n = 2.37). Parameters whose profile does not cross the threshold in one or both directions are non-identifiable: the lower bound for Emax and both bounds for n were non-identifiable. The EC50 lower bound was partially identified. Non-identifiability reflects the sparsity of the in vitro dataset and the absence of a visible plateau in the tested concentration range.

SUPPLEMENTARY FIGURE S3

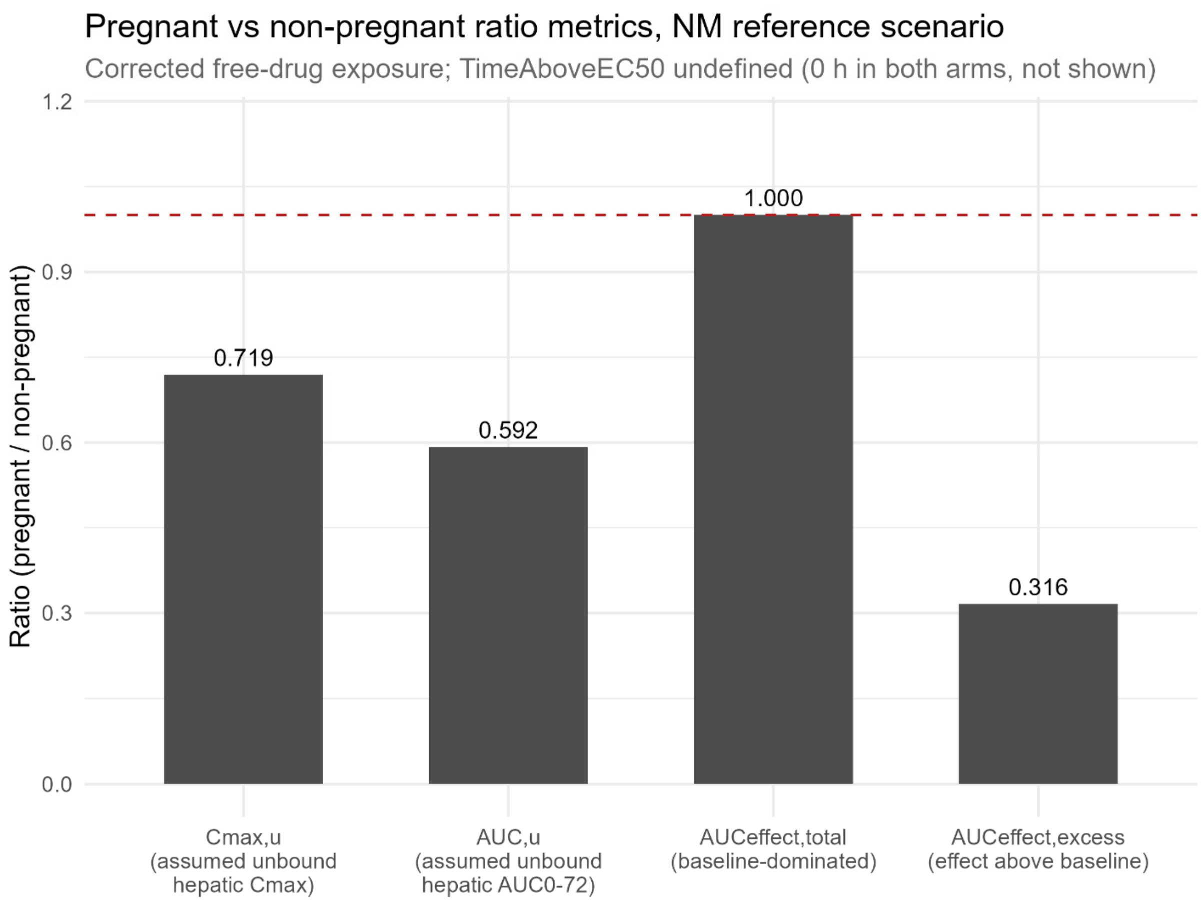

Supplementary Figure S3. Summary of pharmacokinetic and pharmacodynamic ratio metrics (pregnant vs non-pregnant) for the NM reference scenario, under the corrected free-drug exposure. Bars show each metric as the ratio of the pregnant value to the non-pregnant value. The red dashed reference line marks ratio = 1.0. Cmax,u (assumed unbound hepatic Cmax): ratio 0.719; AUC,u (assumed unbound hepatic AUC0-72): ratio 0.592; AUCeffect,total (dominated by the 7200 %-h baseline term): ratio 1.000; AUCeffect,excess (effect above baseline): ratio 0.316; TimeAboveEC50: undefined (zero in both arms). Numerical values are given in Table 3 of the main text. All values are model-derived..

SUPPLEMENTARY FIGURE S4

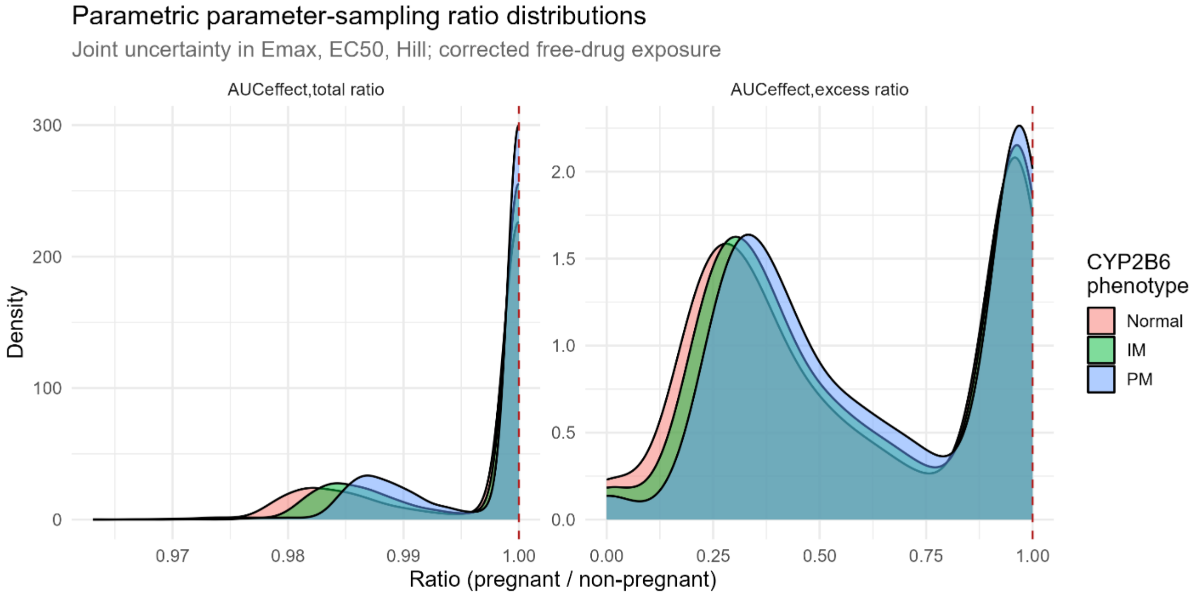

Supplementary Figure S4. Parametric parameter-sampling ratio distributions (pregnant vs non-pregnant) under joint uncertainty in Emax, EC50, and Hill coefficient for NM, IM, and PM CYP2B6 groups, under the fu,vitro-corrected free-drug exposure (Section 2.6 of the main text). The total AUCeffect ratio distribution is concentrated near 1.0 in all three groups (medians approximately 1.000) , reflecting the dominance of the baseline term; the AUCeffect,excess ratio distribution, which isolates the drug-associated signal, is much wider and closer to the identity line (medians 0.312-0.376 across NM/IM/PM; interquartile range approximately 0.13-0.95 in each group), with the interval extending from 0 to approximately 0.97 in all three groups. This is the standalone full-resolution version of Panel D in Figure 3 of the main text. All values are model-derived.

SUPPLEMENTARY FIGURE S5

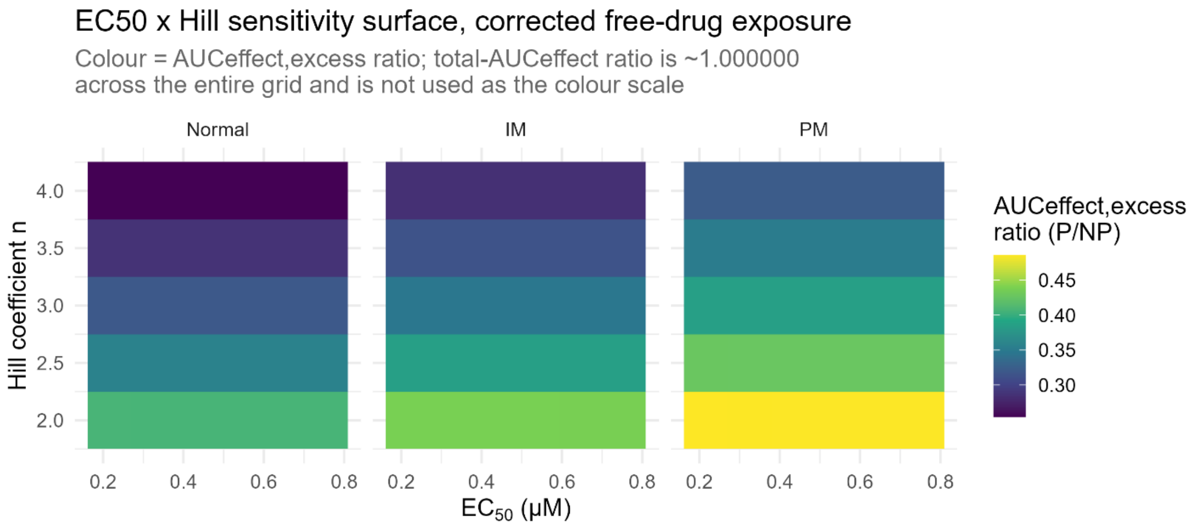

SUPPLEMENTARY FIGURE S5. Two-dimensional EC50 × Hill coefficient sensitivity surface for modelled AUCeffect ratios (pregnant vs non-pregnant), under the fu,vitro-corrected free-drug exposure. Colour scale represents the AUCeffect,excess ratio (the total-AUCeffect ratio is numerically indistinguishable from 1.0 across the entire grid, mean 0.99999996, SD 1.2×10^{-7}, and is not informative as a colour scale). Across 90 parameter combinations (EC50 = 0.216-0.755 µM, the fu,vitro-corrected sensitivity range; Hill coefficient n = 2.0-4.0; Emax held at primary model value), the mean AUCeffect,excess ratio was 0.357 (SD 0.062; range 0.253-0.485), with 100% of combinations producing a ratio below 1.0. The AUCeffect,excess ratio was invariant to EC50 to within numerical precision (identical to at least four decimal places across the full 0.216-0.755 µM range at each fixed Hill value), because both Emax and EC50 cancel algebraically in the pregnant/non-pregnant ratio under the sub-threshold approximation that applies at these exposure levels; only the Hill coefficient determines the ratio. Panels show results for NM, IM, and PM groups separately. All values are model-derived.

SUPPLEMENTARY FIGURE S6

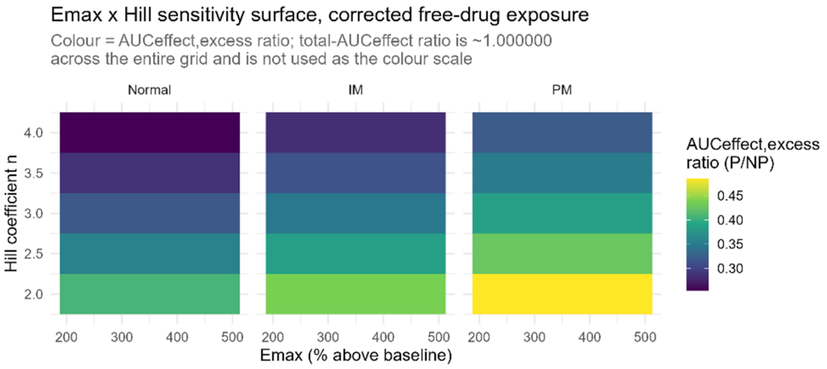

Supplementary Figure S6. Two-dimensional Emax × Hill coefficient sensitivity surface for modelled AUCeffect ratios (pregnant vs non-pregnant), under the corrected free-drug exposure. Colour scale represents the AUCeffect,excess ratio, for the same reason given in Figure S5. Across 195 parameter combinations (Emax varied from 200 to 500 percentage points above baseline; Hill coefficient n = 2.0-4.0; EC50 held at primary model value), the mean AUCeffect,excess ratio was 0.357 (SD 0.062; range 0.254-0.485), with 100% of combinations producing a ratio below 1.0. All values are model-derived.

SUPPLEMENTARY FIGURE S7

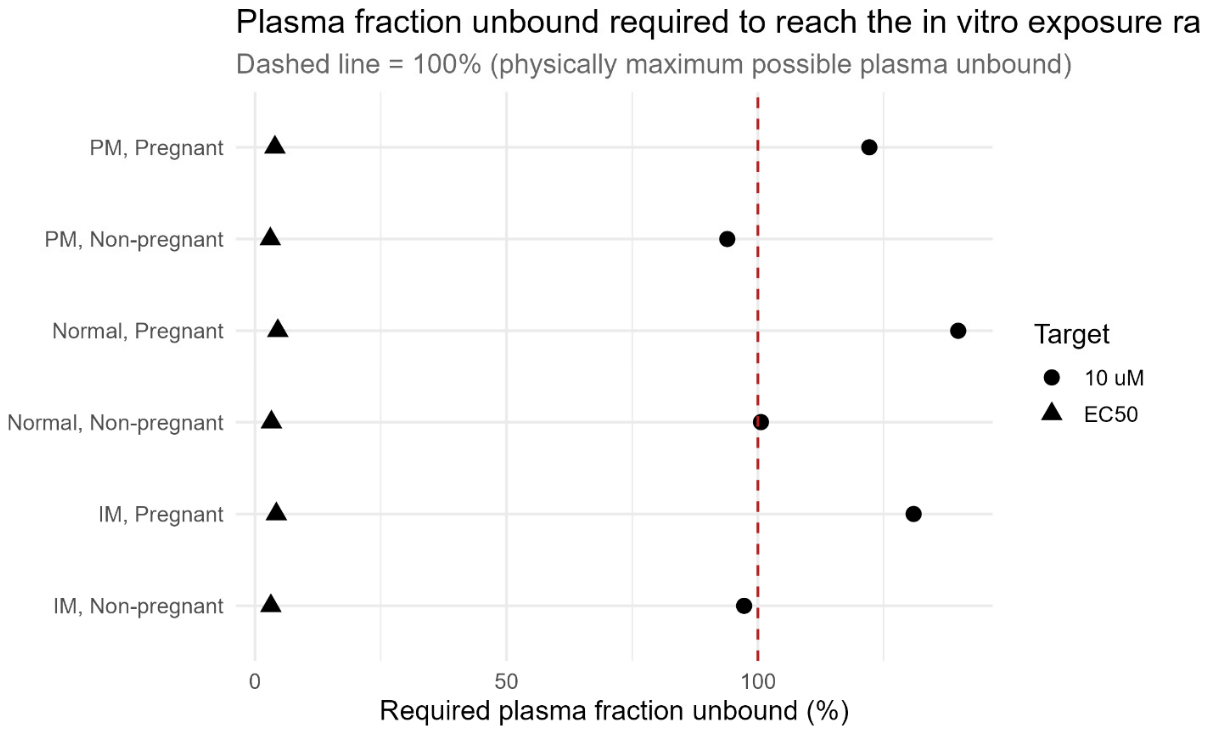

Supplementary Figure S7. Plasma fraction unbound required, under the free-drug equilibrium approximation, for predicted total plasma Cmax to yield an unbound hepatic concentration of 10 µM (circle) or the fu,vitro-corrected reference EC50 (0.324 µM; triangle), across all six scenarios. This diagnostic is independent of any assumed fu,p value. Values exceeding 100% (dashed line) indicate the target is mathematically unreachable at any physiologically possible plasma protein binding. All values are model-derived..
